# Role of the Blood-Brain Barrier in the Formation of Brain Metastases

**DOI:** 10.3390/ijms14011383

**Published:** 2013-01-11

**Authors:** Imola Wilhelm, Judit Molnár, Csilla Fazakas, János Haskó, István A. Krizbai

**Affiliations:** Institute of Biophysics, Biological Research Centre of the Hungarian Academy of Sciences, Temesvári krt. 62, Szeged H-6726, Hungary; E-Mails: wilhelm.imola@brc.mta.hu (I.W.); molnar.judit@brc.mta.hu (J.M.); fazakas.csilla@brc.mta.hu (C.F.); hasko.janos@brc.mta.hu (J.H.)

**Keywords:** blood-brain barrier, cerebral endothelial cell, tight junction, paracellular transmigration, astrocyte, central nervous system, metastasis, breast cancer, lung cancer, melanoma

## Abstract

The majority of brain metastases originate from lung cancer, breast cancer and malignant melanoma. In order to reach the brain, parenchyma metastatic cells have to transmigrate through the endothelial cell layer of brain capillaries, which forms the morphological basis of the blood-brain barrier (BBB). The BBB has a dual role in brain metastasis formation: it forms a tight barrier protecting the central nervous system from entering cancer cells, but it is also actively involved in protecting metastatic cells during extravasation and proliferation in the brain. The mechanisms of interaction of cancer cells and cerebral endothelial cells are largely uncharacterized. Here, we provide a comprehensive review on our current knowledge about the role of junctional and adhesion molecules, soluble factors, proteolytic enzymes and signaling pathways mediating the attachment of tumor cells to brain endothelial cells and the transendothelial migration of metastatic cells. Since brain metastases represent a great therapeutic challenge, it is indispensable to understand the mechanisms of the interaction of tumor cells with the BBB in order to find targets of prevention of brain metastasis formation.

## 1. Introduction

Brain metastases constitute a significant part of intracranial tumors. Only in the United States, about 170,000 metastatic brain tumors are diagnosed annually [1], whereas primary tumors represent 17,000 new cases/year. The majority of brain metastases originate from lung cancer (40%–50%), breast cancer (15%–25%) and malignant melanoma (5%–20%) [2]. Among these tumors, melanoma is the one which metastasizes to the brain with one of the highest frequencies: brain metastases are diagnosed in 40%–50% of the cases, which, after autopsy, increase with an additional 30%–40%. Melanoma is diagnosed in six–40 cases/100,000 inhabitants/year, causing 4% of all skin cancers and being responsible for 74% of skin cancer deaths. Of melanoma brain metastases, 49% are intraparenchymal, 22% are leptomeningeal and 32% are dural [3]. The presence of a brain metastasis represents a very poor prognosis for the patient, the five-year survival rate being below 10%. The number of diagnosed melanoma cases is constantly increasing [4].

Breast cancer is the second most common type of cancer and worldwide causes more than half a million deaths annually. Breast cancer metastases to the central nervous system (CNS) include the clinically distinct situations of multiple brain metastases (78%), solitary brain metastasis (14%) and leptomeningeal metastases (8%) [5]. CNS metastases occur in 10%–16% of stage IV patients, while they are found in 30% of patients in autopsy series [5].

The most common cause of brain metastases is lung cancer. Interestingly, small cell carcinomas, which are only 20% of all lung cancers, account for 50% of brain metastases from lung cancer. About 10% of the patients having small cell lung carcinoma initially present with brain metastases. The two-year cumulative risk rises to ≥50%, and CNS metastases are found in up to 65% of patients at autopsy. The median survival time after brain metastasis diagnosis is four to five months [6].

Formation of metastases in distant organs involves blood or lymphatic transport of cancer cells. Since the CNS lacks a lymphatic system, the only possibility for cancer cells to reach the brain is via the blood stream. Brain metastases can be formed both in the parenchyma and the meninges. Leptomeningeal metastases resulting from solid tumors occur late and usually coexist with CNS parenchymal disease. Metastatic cells invading the CNS parenchyma, however, have to pass the blood-brain barrier (BBB).

## 2. The Blood-Brain Barrier

### 2.1. Cellular Structure of the BBB

**Figure 1:**
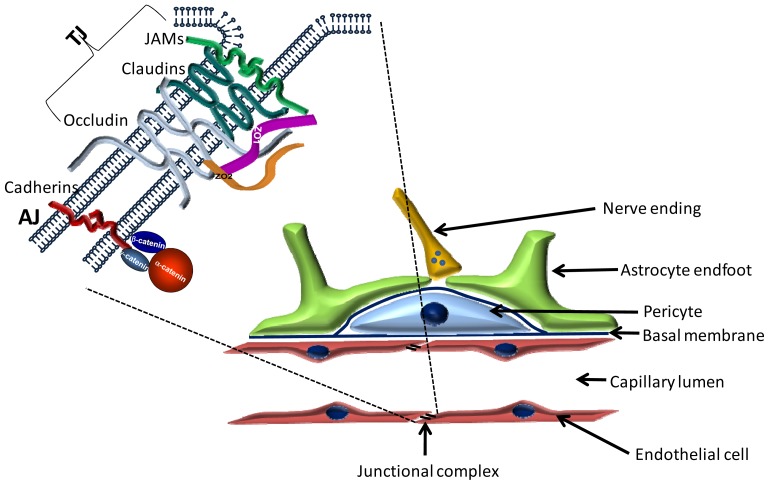
Schematic structure of the blood-brain barrier. Cerebral endothelial cells—coming in contact with pericytes and astrocytes—form the morphological basis of the blood-brain barrier. Endothelial cells are interconnected by a continuous line of tight junctions. The insert shows the molecular structure of the junctional complex. TJ = tight junction; AJ = adherens junction.

The blood-brain barrier (BBB) is located at the level of cerebral capillaries in the forefront of the defense line of the CNS and restricts the free movement of solutes and cellular elements between the systemic circulation and neuronal tissue. The BBB is involved in the pathogenesis of a large number of CNS disorders [7,8]. The most important cellular elements of the BBB are endothelial cells, astrocytes and pericytes forming the neurovascular unit (Figure 1).

#### 2.1.1. Endothelial Cells

Endothelial cells lining brain capillaries are thin, flat cells interconnected by tight junctions [9] and characterized by a high number of mitochondria [10] and low number of caveolae [11]. The contact region of brain endothelial cells is usually overlapping, and the apical region of cell membranes is interconnected by a continuous line of tight junctions limiting the free transport of different solutes and cellular elements. Cerebral endothelial cells share common features with other endothelia (presence of factor VIII, high alkaline phosphatase and *γ*-glutamyl transpeptidase activity, uptake of acetylated-low density lipoprotein) and epithelia as well (high transendothelial electrical resistance (TEER), continuous line of tight junctions, low level of pinocytosis), these latter being indispensable for the barrier function.

#### 2.1.2. Pericytes

Pericytes are located in the duplication of the basement membrane, in close contact with endothelial cells. Even gap junctions have been described between the two cell types [12]. In the rat brain, pericyte coverage of the capillaries varies between 22% and 32% [13] and the ratio of pericyte/endothelial cells is about 1/3–5 [14]. Pericytes are contractile cells able to synthesize a plethora of biologically active substances. Although the exact function of pericytes in the formation and function of the BBB is insufficiently understood, they can participate in the regulation of blood flow, endothelial proliferation, angiogenesis or inflammatory processes. Absence of pericytes leads to endothelial hyperplasia, abnormal vasculogenesis [15] and increased BBB permeability [16]. Pericyte-endothelial cell interactions were found to be critical in the regulation of the BBB during development [17].

#### 2.1.3. Astrocytes

Astrocytes play a crucial role in the induction of blood-brain barrier properties (reviewed in: [18,19]). Astrocytic endfeet nearly completely ensheath the capillary walls, thereby covering not only endothelial cells, but also the intimately associated pericytes [20]. The coverage is not complete, allowing a direct contact of nerve endings with the basal membrane [21,22]. Astrocytic endfeet express a high level of several specific proteins at their capillary side, like glucose transporter 1, P-glycoprotein (P-gp), aquaporin-4, connexin-43 and Kir 4.1 K^+^ channel.

#### 2.1.4. Other Cells of the Neurovascular Unit

In the adult mammalian brain, neurons are usually not in direct contact with cerebral endothelial cells. So far, it is unclear whether there are signals from endothelial cells to neurons and *vice versa,* which could be important for brain homeostasis or for neuronal function. Such communication could be accomplished by the presence of neurotransmitter receptors on endothelial cells, as observed previously [23].

Microglia is another cell type that is in close contact with cerebral vessels. The role of microglia in the neurovascular unit is still poorly understood, and sometimes controversial. They may potentiate BBB damage during neuroinflammation [24]; however, a beneficial effect has also been reported in response to ischaemic brain injury [25].

#### 2.1.5. The Basement Membrane

The basement membrane is a specialized extracellular matrix covering endothelial cells from the outside. Its main protein components include collagen (especially type IV), fibronectin, laminin, tenascin and proteoglycans [11]. The extracellular matrix serves as an anchor for endothelial cells through the interaction between laminin and other matrix proteins and endothelial integrin receptors [26]. Besides signaling events [27], the cell-matrix interaction also modulates TJ protein expression [28]. Furthermore, the basal membrane plays an important role in cell adhesion and migration and may form a barrier for macromolecular and cellular migration. Moreover, the basal membrane was shown to be essential for the survival of cancer cells during brain colonization [29].

### 2.2. Molecular Structure of the BBB

Transport across the brain endothelium is strictly limited through a four-fold defense line (for review see: [30]): paracellular barrier (represented by interendothelial junctions); transcellular barrier (assured by the low level of endocytosis and transcytosis); enzymatic barrier (including acetylcholinesterase, alkaline phosphatase, γ-glutamyl transpeptidase, monoamine oxidases and drug metabolizing enzymes); and efflux transporters (ABC-B1, -C1, -C4, -C5 and -G2). Small gaseous molecules, such as O_2_ and CO_2_, can freely diffuse through the lipid membranes, and this is also a route of entry for small lipophilic agents, including barbiturates, nicotine and ethanol. However, specific blood-to-brain influx transport systems exist to supply nutrients, like glucose, amino acids and nucleotides, which cannot freely diffuse to the brain.

The paracellular permeability is mainly regulated by the tight junctions (TJs) between endothelial cells (Figure 1). Key components of the tight junctions are the transmembrane proteins, which form three protein families. These are the four transmembrane proteins (occludin, claudins, tricellulin/marvelD2, marvelD3), which are perhaps the most important from the point of view of paracellular permeability, molecules belonging to the immunoglobulin superfamily (JAM—Junctional adhesion molecule; CAR—Coxsackie and adenovirus receptor; ESAM—Endothelial cell-selective adhesion molecule) and non-immunoglobulin-like molecules with a single transmembrane domain (CRB3—Crumbs homolog 3; Bves—Blood vessel epicardial substance). Best characterized in cerebral endothelial cells are occludin, claudins and JAMs [31].

#### 2.2.1. Transmembrane TJ Proteins

##### 2.2.1.1. Occludin

Occludin, the first identified transmembrane TJ protein [32], is a 65 kDa molecule. It is characterized by four transmembrane regions, two extracellular loops, a shorter N-terminal and a longer C-terminal cytoplasmic domain. The two extracellular loops are rich in tyrosine and glycine, playing a role in sealing the junctions [33,34], while the C-terminal region is important in the interaction with other junctional proteins.

##### 2.2.1.2. Claudins

Claudins, first described by Furuse *et al.* [35], are small proteins (20–27 kDa), which show a similar membrane topology to occludin (short N-terminal cytoplasmic region, two extracellular loops, short C-terminal tail); however, there is no sequence homology between them. Interactions of claudins are largely determined by the C-terminal intracellular region, which contains PDZ binding domains. Furthermore, claudins are able to form homophylic interactions as well needed for the formation of tight junction strands [36]. The principal claudin in brain endothelial cells is claudin-5, but other claudins (especially claudin-1, -3 and -12) have also been detected [37]. The exact role of individual claudins is not known; absence of claudin-5 leads to a selective opening of the BBB to molecules smaller than 800 Da [38]. Claudins play an important role in several pathologies, including cancer [39].

##### 2.2.1.3. Immunglobulin-like Molecules

Junctional adhesion molecules (JAMs) are single-span molecules belonging to the immunoglobulin superfamily characterized by homophilic binding and two extracellular loops, first described by Martin-Padura *et al.* [40]. Brain endothelial cells express mainly JAM-1 (JAM-A) and JAM-3 (JAM-B) [41], but also JAM-C. They are involved in the extravasation of leukocytes. Endothelial cells also express ESAM (endothelial cell-selective adhesion molecule), another immunoglobulin-like molecule localized to the TJs. JAM-C and ESAM have been shown to promote melanoma lung metastasis formation [42,43].

##### 2.2.1.4. Other Transmembrane Proteins of the TJs

In epithelial cells several additional components of the TJs have been identified, the expression/role of which has not been characterized in endothelial cells. These molecules include tricellulin, CAR, marvelD3 and Crumbs homolog 3 (CRB3).

#### 2.2.2. Peripheral Proteins of Tight Junctions

##### 2.2.2.1. PDZ Domain Containing Proteins

###### 2.2.2.1.1. Zonula Occludens (ZO) Proteins

To our present knowledge, there are three members of the zonula occludens family: ZO-1 [44], ZO-2 [45] and ZO-3 [46]. ZO-3 seems to be less important in the formation of endothelial tight junctions [47]. Common structural features of the ZO family include three PDZ domains in the N-terminal region, a SH3 (Src homology 3) domain and an enzymatically inactive GUK (guanylate kinase) domain. ZO proteins are important scaffold proteins, but are essential in signaling processes as well [48,49].

###### 2.2.2.1.2. Other Proteins Containing PDZ Domain

The cytoplasmic plaque of the TJs contains other PDZ domain containing proteins as well. These include AF6/afadin, MUPP1 (multi-PDZ-domain protein), MAGI (membrane associated guanylate kinase inverted) -1, -2 and -3 and PAR-3 and -6. These proteins also bind other proteins of the junctional complex, but their role in the regulation of endothelial TJs has not been characterized yet.

##### 2.2.2.2. Plaque Proteins without PDZ Domain

Besides PDZ domain proteins, the junctional plaque contains cingulin, its homologue paracingulin/JACOP and a large number of signaling molecules.

## 3. Mechanisms of Interaction of Tumor Cells with Brain Endothelial Cells

The BBB, which represents the tightest endothelial barrier in the organism, forms an obstacle for the traffic of not only solutes, but cells as well. Penetration of cells into the CNS is highly limited by the BBB; therefore, it is surprising that some cancer types give metastases preferentially to the brain. This phenomenon draws the attention to the possibility that besides being a barrier, the BBB may have a supportive role in the metastasis formation as well. Indeed, cerebral endothelial cells can actively take part in the transmigration process and may even facilitate the penetration of metastatic cells or can provide an ideal milieu for transmigrated cells to survive in their close proximity. This can be due to the fact that after crossing the barrier, metastatic cells are protected from the immune surveillance of the organism and cellular components of the BBB may release substances favorable for metastasis growth, as discussed in the forthcoming chapters.

Despite an impressive amount of data regarding motility and migration of cancer cells, information about the mechanisms involved in the migration of cancer cells across endothelial barriers is limited. This is especially true for the BBB. The process of transendothelial migration has been intensively studied using immune cells. Although the steps of transmigration (rolling, adhesion and transmigration/diapedesis) may show some similarities, due to different physiological, molecular and mechanical characteristics of immune and metastatic cells, there may be significant differences [50].

### 3.1. Morphological Aspects

Arrest of tumor cells was found to take place at the level of capillaries and postcapillary venules, where the diameter of the vessels is comparable to those of the metastatic cells, predominantly at vessel branches [51,52].

The extravasation process of tumor cells seems to have organ-specific elements. Tumor cells need a significantly longer time to extravasate into the brain than into other organs. In the brain, reaching an extraluminal position was found to take two days in case of lung and two-seven days in case of breast cancer cells [51,53]. Kienast *et al.* [52] have observed that melanoma and lung cancer cells have extravasated up to 14 days; however, cancer cells that later proliferated into macrometastases had left the blood vessel by the third day. The diapedesis itself is probably rapid [53], but arrested cancer cells need to survive within the brain vasculature for a significantly longer time than in other organs [51]. This seems to be a rate limiting step only in the brain. Intravascular proliferation preceding transendothelial migration seems to characterize only some cell lines with high affinity for the brain [51].

Cancer cells arrested in the brain vasculature initially assume an elongated shape and, later, round up and stretch the vessel walls [51]. During the transmigration process, tumor cells show intra- and extra-vascular parts with a narrowing at the level of the vascular wall, suggesting that the transmigration takes places through holes in the endothelium. In addition, tumor cells produce extensions and retractions of the extravascular protrusions, indicating that the transmigration process is a dynamic one [52].

The contribution of endothelial cells seems to be organ-specific as well: while in the lung and liver, endothelial cells migrate onto the surface of the tumor cells, in the brain, endothelial cells do not cover tumor cells; instead, a retraction of endothelial cells was observed [53]. It is a question of debate whether tumor cells leave the endothelium intact or destruct the vessel wall during diapedesis. Breast cancer cells were found to transmigrate at sites of discontinuity of the vessel wall, but no endothelial apoptosis or hypoxia was observed [51]. Using an *in vitro* system, we have observed that melanoma cells damaged the integrity of the brain endothelial monolayer, induced endothelial apoptosis and decreased the transendothelial electrical resistance [54]. It was suggested, however, that the barrier can repair after passage of metastatic cells [55]. On the other hand—although extravasation of single cells is the dominant mechanism of brain metastasis formation—in some cases, intravascular proliferation of tumor cells may lead to the complete obstruction of the vessel and, finally, to the disruption of the BBB [56].

After diapedesis, endothelial cells are indispensable for the proliferation of metastatic cells, formation of the tumor vasculature (by angiogenesis or vessel cooption) and of the blood-tumor barrier. Proliferation of metastatic cells in the brain was only observed when transmigrated metastatic cells remained in close contact to the basolateral side of endothelial cells, in a position similar to pericytes [52]. Moreover, breast cancer cells extravasated into the brain were shown to get aligned along the blood vessels, on the extraluminal side [56]. According to our observations, after completing the transendothelial migration process, melanoma cells continue their movement beneath the endothelial cell layer [54]. Some perivascularly located single metastatic cells (or clusters up to three) can remain dormant for longer periods [52]. These cells can constitute a source for the formation of new metastases.

The long-lasting close contact of extravasated tumor cells with the endothelium may have two reasons. First, the vascular basement membrane supports the growth of the metastasis prior to the formation of the tumor vasculature [29]. This is the so-called vessel cooption, which seems to be characteristic to breast cancer and melanoma, but not lung cancer cells [29,52,57]. In contrast to melanoma and breast cancer, lung cancer metastases were shown to present early angiogenesis [52].

Second, cancer cells might benefit from the protection of the BBB against anti-tumoral immune cells and chemotherapeutics. It is a question of debate whether the vascular network of the metastases exhibits altered permeability. The blood-tumor barrier was suggested to remain intact in small metastases, its integrity altering only when larger lesions are formed [55]. Others state that in breast cancer metastases, the blood-tumor barrier has an increased permeability, which poorly correlates with the size of the lesion. However, the barrier remains sufficiently intact to impair drug delivery [58].

**Figure 2:**
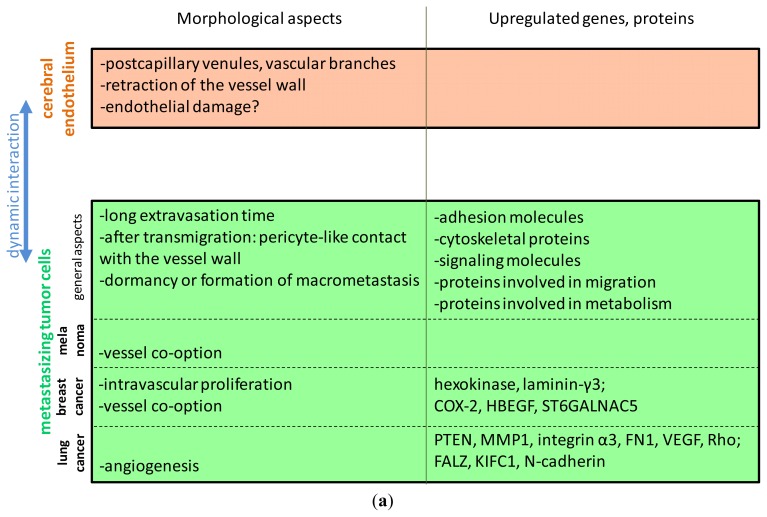

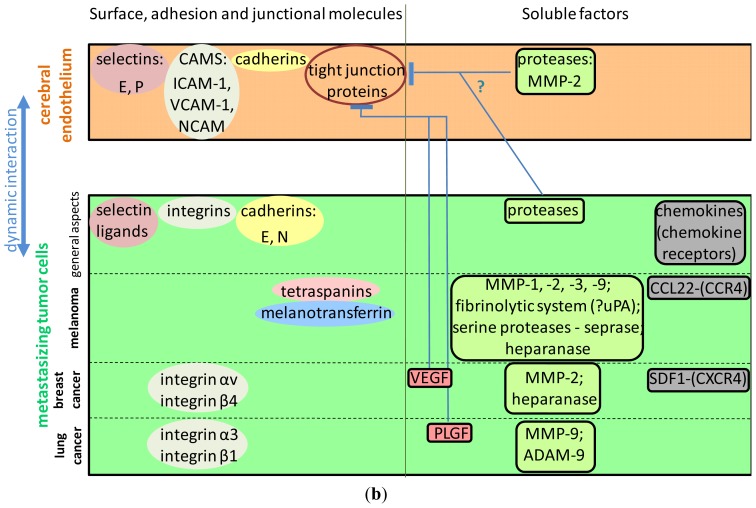
Mechanisms of interaction between tumor cells and the brain endothelium. (**a**) Morphological aspects and upregulated genes and proteins involved in brain metastasis formation of melanoma, breast and lung cancer; (**b**) Molecular mechanisms. Several adhesion molecules were shown to be involved in the adhesion of tumor cells to the brain endothelium. Proteases and other soluble factors secreted by tumor cells may induce the disruption of the tight junctions.

The morphological aspects of the interaction of tumor cells with the brain endothelium are summarized in Figure 2a.

### 3.2. Selectively Expressed Genes and Proteins in Brain Metastatic Cells

One of the crucial issues in the goal to prevent or reduce the number of metastases is the understanding of molecular mechanisms of organ-specific metastases. A key determinant of the site of metastasis formation is the gene or protein expression profile of metastatic cells with affinity to a specific organ. Specific gene expression combinations may determine the ideal conditions in which the tumor cell can survive and divide, thus determining preferential target organs. On the other hand, in the case of the brain—which has a specific vasculature lined by BBB endothelial cells—these expression profiles may determine the interaction of metastatic cells with the endothelium as well.

In one of the first attempts to identify differentially expressed genes, the authors compared gene expression of primary lung adenocarcinoma (a frequent subtype of non-small cell lung carcinoma) with brain metastases originating from these tumors. They have found considerable differences: of 23,040 genes tested, 244 showed a different expression level, including genes coding for plasma membrane proteins, cellular antigens and cytoskeletal proteins, which may modulate cell-cell interactions [59].

Brain metastases of lung adenocarcinoma were evaluated in another study as well, by comparing the gene expression profile of metastatic brain tumors originating from lung adenocarcinoma with that of healthy lung. Using cDNA microarray technology, 1,561 differently expressed genes were found. The overexpression of certain genes associated with invasion and metastasis (PTEN, MMP1), adhesion (integrin α_3_ and fibronectin1), angiogenesis (VEGF) and cell migration (Rho GTPase) was validated by real-time PCR [60].

A correlation analysis of frozen samples from 142 patients diagnosed with non-small cell lung carcinoma revealed three genes that were associated with brain metastases. Expression of these three genes was predictive for brain metastases: FALZ (fetal Alzheimer antigen), KIFC1 (kinesin family member involved in cell proliferation) and N-cadherin. While the first two may play a role in the growth of metastases in a neural environment, expression of N-cadherin may play a role in the interaction of metastatic cells with brain endothelial cells as well, which are also able to express N-cadherin [61].

In another study, a multidimensional proteomic analysis was used to examine the protein expression profiles of breast cancer cells, by comparing the parental cell line and its brain or bone homing variants. More than 300 proteins were found to be uniquely regulated in brain metastatic breast cancer cells, most of which are involved in cellular metabolism and cell stress response. These data reflect the adaptation of the tumor cells to aerobic glycolysis, which is more favorable in the brain environment [62].

Brain and bone metastases of breast cancer cells were compared by Klein *et al.* [63] as well. 73 differentially expressed genes were found, with 51 genes having significantly higher expression in brain metastases. Among the genes overexpressed in the brain, several encode proteins involved in translation, metabolism, signal transduction and transport, but there are adhesion molecules as well, which may play a role in the interaction of metastatic cells with the cerebral endothelium.

Investigating breast cancer brain metastases, Palmieri *et al.* [64] have revealed downregulation of six genes and upregulation of two: hexokinase and laminin-*γ*3.

Finally, in a genomewide comparative study, the authors have found 243 genes over- or under-expressed in the brain metastatic population of breast cancer cells. Further analysis identified COX2, the epidermal growth factor receptor ligand HBEGF, and the α2,6-sialyltransferase ST6GALNAC5 as important determinants of transmigration of breast cancer cells through the BBB. While COX2 and HBEFG were also found in lung metastases, ST6GALNAC5 was found to be specific to brain metastases. ST6GALNAC5 is a brain-specific sialyltransferase and may directly influence breast cancer cell adhesion to cerebral endothelial cells [65].

Summarizing the results, one can observe that the different studies have found some members of gene classes involved in adhesion and proliferation to be increased in brain metastatic tumor cells (Figure 2a). It is noteworthy, however, that there is a low overlap between the different sets of genes identified. Which of these are involved in the interaction of tumor cells and brain endothelial cells still needs to be elucidated.

### 3.3. Transmigration Routes: Role of the Proteins of the Tight Junctions

Transendothelial migration of cells can occur by two routes: the paracellular pathway (through the interendothelial junctions) and the transcellular one (through single endothelial cells). Leukocytes are able to use both routes in the brain endothelium as well [66,67], and the molecular mechanisms involved in the paracellular and transcellular migration pathways of leukocyte diapedesis have been intensively studied (reviewed in: [68,69]). Much less is known about the transmigration routes of tumor cells, especially in the brain.

Paracellular transmigration of metastatic cells is possible only with the involvement of endothelial tight junctions and junctional proteins. In a recent study using an *in vitro* model of the BBB, we have observed that different types of melanoma cells were able to reduce transendothelial electrical resistance (TEER), which is a widely used indicator of junctional integrity [54]. Moreover, not only the presence of metastatic cellular elements, but their conditioned media, was also able—although to a lesser extent—to reduce TEER. Immunfluorescence studies revealed that the disruption of the TJs stands at the molecular basis of the increased permeability, reflected by a discontinuous staining of claudin-5 and ZO-1. After longer coculture times, melanoma cells were able to make holes in the endothelial monolayer, which could be used by other metastatic cells. The mechanisms by which metastatic cells are able to disrupt TJs are incompletely understood; however, proteolytic processes probably play an important role.

On the other hand, it is not excluded that tumor cells might also use the transcellular pathway; especially as brain endothelial cells are interconnected by tight junctions, which seal the intercellular way of transport. So far, transcellular migration of tumor cells has only been described in the case of intravasation of breast cancer cells into an artificial vascular network prepared from calf pulmonary artery endothelial cells [70].

### 3.4. Surface Molecules Mediating Different Steps of Tumor Cell Extravasation in the Brain

Adhesion and junctional molecules involved in tumor cell-endothelial cell interaction and metastasis formation are weakly characterized and even less is known about brain endothelial-specific mechanisms. It is well known that tumor cells are able to partly mimic the molecular mechanisms of leukocyte-endothelial interaction occurring during inflammation. The steps of transendothelial migration of leukocytes and tumor cells are principally the same, *i.e.*, rolling, adhesion and diapedesis; however, on the molecular level, the transmigration process of tumor cells has been much less described [50]. In this interaction, surface molecules of both endothelial and tumor cells might be involved (Figure 2a). Moreover, tumor cells were shown to indirectly use the adhesion molecules of leukocytes and platelets, by attaching to them and using them as bridges to the endothelium, making the molecular aspect even more complex [71,72]. In this chapter, we review the surface molecules of both endothelial and tumor cells involved in the interaction of tumor cells with brain endothelial cells.

#### 3.4.1. The Role of Selectins and Selectin Ligands

Selectin-dependent mechanisms mediate tethering and rolling of leukocytes during the first steps of extravasation. Cancer cells, similar to leukocytes, express selectin ligands, which may play an important role in their adhesion to endothelial cells. Selectin-dependent mechanisms are also important in the interaction of tumor cells with platelets and leukocytes, which facilitate the attachment of tumor cells to the vessel wall. The role of selectins in brain metastasis formation is largely uncharacterized. Anti-E-selectin antibodies were shown to partly inhibit adhesion of primary prostate carcinoma cells to brain endothelial cells [73]. Moreover, heparin—which inhibits not only coagulation, but selectin-mediated interactions as well—was shown to delay melanoma brain metastasis formation. Therefore, it has been suggested that heparin might have an anti-metastatic neuroprotective role, which might depend on selectins [74].

#### 3.4.2. Integrins Involved in the Interaction of Tumor Cells with the Brain Endothelium

Integrins are heterodimeric proteins consisting of a α and a β subunit that mediate cell-cell and cell-extracellular matrix interactions. They facilitate the firm adhesion of leukocytes to the endothelium. Several integrins were shown to be involved in cancer progression, metastasis formation, transendothelial migration of tumor cells and angiogenesis in different metastatic sites. Activation of integrin α_v_β_3_ was observed to support efficient brain metastatic growth of breast cancer cells through continuous upregulation of VEGF, without influencing the growth of primary lesions [75]. Moreover, intetumumab, an anti-integrin α_v_ monoclonal antibody, prevents brain metastasis formation of breast cancer cells [76]. Interaction of breast cancer cells with the brain endothelium might also be dependent on integrin β_4_. Integrin β_4_ signaling is involved not by directly promoting the adhesion of tumor cells to the endothelium, but by enhancing the HER2-dependent expression of VEGF, which induces the disruption of interendothelial junctions [77]. In the case of non-small cell lung cancer, the interaction of integrin α_3_β_1_ and laminin was suggested to play an important role in brain metastasis formation [78]. Blockade or loss of β_1_ integrin in mammary carcinoma cells prevents metastasis establishment and growth of the tumor cells in the CNS [29].

#### 3.4.3. The Role of the Immunglobulin Superfamily of Cell Adhesion Molecules

Endothelial cells express several adhesion molecules belonging to the immunoglobulin (Ig) superfamily, including members of the intercellular adhesion molecules (ICAMs), vascular-cell adhesion molecule (VCAM-1), platelet-endothelial-cell adhesion molecule (PECAM-1) and junctional adhesion molecules (JAMs). These are essential in immune response and inflammation, but some of them were also shown to be involved in the interaction of vascular endothelial and tumor cells and formation of metastases. Anti-VCAM-1 antibodies were shown to partly inhibit adhesion of primary prostate carcinoma cells to brain endothelial cells [73]. Moreover, establishment of pulmonary melanoma metastases was found to be followed by increases in VCAM-1 expression in organs frequently affected by melanoma metastases, including the brain [79]. In a recent paper, ICAM-1 and VCAM-1 were shown to play a crucial role in polychlorinated biphenyl-mediated enhancement of brain metastasis formation of lung carcinoma cells [80]. NCAM was shown to be highly expressed in the primary tumors of brain metastasis patients [81]; However, brain metastases were found to lose NCAM expression [82]. This may suggest that NCAM is important for the formation of brain metastasis (e.g., for transendothelial migration of tumor cells into the brain) and not for the survival or proliferation of metastatic cells in the brain environment.

#### 3.4.4. Cadherins

Cadherins are Ca^2+^-dependent cell adhesion molecules fundamental in tissue organization. Cadherin dysfunction is involved in tumor progression and metastasis formation. Loss of expression of E-cadherin induces epithelial-mesenchymal transition in carcinoma cells, which initiates an increase in cell motility and metastasis formation. In metastatic lesions, a re-expression of E-cadherin has been observed, which plays an important role in the proliferation of tumor cells at the metastatic site. Correspondingly, metastatic brain tumors were shown to express high levels of E-cadherin [83–85], while low expression of E-cadherin in primary non-small cell lung carcinomas was shown to correlate with increased risk for the development of brain metastasis [86]. In non-small cell lung carcinomas, the expression level of N-cadherin was observed to be highly predictive of brain metastasis formation [61]. Since transendothelial migration of melanoma cells through human lung microvascular endothelial cells was found to involve N-cadherin-mediated adhesion [87], a similar mechanism is possible in the case of brain endothelial cells as well. N-cadherin was shown to be recruited to the contact sites between transmigrating melanoma cells and pulmonary endothelial cells, followed by its Src kinase-mediated phosphorylation and dissociation of β-catenin from N-cadherin [88].

#### 3.4.5. Tetraspanins

Several members of the tetraspanin superfamily, including CD9, CD81 and CD151, might localize at the tumor cell-endothelial cell heterologous contact area. Among these, endothelial CD9 was shown to actively redistribute to points of melanoma insertion, and anti-CD9 antibodies were found to inhibit migration of melanoma cells through HUVEC monolayers [89]. CD151-null mice showed a markedly diminished number of experimental lung metastasis after injection of Lewis lung carcinoma or B16F10 melanoma cells [90]. Similar mechanisms might be involved in the transmigration of tumor cells through the BBB as well.

#### 3.4.6. Melanotransferrin

Melanotransferrin has been identified as a surface molecule on melanoma cells. Expression of melanotransferrin was found to correlate with increased transmigration of melanoma cells through the BBB, while blocking melanotransferrin significantly reduced transmigration. In mediating the effect of melanotransferrin, the plasmin-metalloproteinase system could be involved [91].

### 3.5. Soluble Factors Affecting Brain Metastasis Formation

Tumor cells secrete several factors that may enhance their migration through the brain endothelium (Figure 2b). Brain-derived soluble factors may also play a significant role in the formation of brain metastases. This may be true especially in the case of melanoma, where the high proportion of brain metastases may be due to a “homing” influence [92], because melanocytes and the nervous system share a common embryologic (ectodermal) origin.

#### 3.5.1. Neurotrophins

Neurotrophins (NTs: NGF, BDNF, NT-3 and NT-4/5) are growth factors promoting neuronal survival, differentiation and cell death. Invasive and survival properties of CNS-metastatic melanoma cells were shown to be dependent on the presence of particular NTs that can be secreted by different cell types within the CNS. Brain metastatic melanoma cells may induce the production of brain NTs that aid in the survival and invasion of melanoma cells in the CNS [93]. In addition, NGF enhances the production of extracellular matrix-degradative enzymes in melanoma cells [92].

In the brain, NTs and NT receptors are known to be expressed by neurons and astrocytes. However, recent data suggest that NT release and signaling in the CNS may not be restricted to these two cell types. Murine and rat brain endothelial cell lines were shown to express NGF, BDNF and two NT receptors (TrkA and p75) [94,95]. Furthermore, pericytes are also able to express NGF and BDNF [96]. Therefore, one can speculate whether NTs play a role not only in the survival and proliferation of metastatic melanoma cells in the CNS, but in their migration through the BBB as well.

#### 3.5.2. Chemokines

An important role in the formation of brain metastases seems to have chemokines and their receptors as well. In a recent comprehensive study, melanoma cells were shown to express a whole set of chemokine receptors, including CCR3, CCR4, CXCR3, CXCR7, CX3CR1 and membrane CX3CL1. Among these receptors, the expression of CCR4 was found to be significantly higher in a brain metastatic melanoma variant. Activation of CCR4 by its ligand CCL22 induced a specific Akt phosphorylation pattern, suggesting that specific signaling may be related to brain metastasis formation [97]. Moreover, brain-derived soluble factors were able to upregulate the expression of CCR3 and CCR4 in melanoma cells and enhanced the transmigration of melanoma cells through a monolayer of cerebral microvascular endothelial cells [98], further indicating that the brain microenvironment is not only important for the growth of already formed brain metastases, but also for the transmigration of melanoma cells through the BBB. Chemokines seem to be important in the brain metastasis formation of breast cancer cells as well, since the CXCR4/SDF1 signaling pathway was shown to have a decisive role in the migration of breast cancer cells through brain endothelial monolayers [99].

#### 3.5.3. Vascular Endothelial Growth Factor and Its Receptors

Being a key modulator of angiogenesis, VEGF is a well-established factor in the growth of metastases. However, VEGF secreted by extravasating tumor cells may be involved in the transmigration process as well, mainly by enhancing the permeability. In breast cancer cells, HER2 increases VEGF protein production, which induces the disruption of interendothelial junctions [77]. In addition, VEGF was shown to increase the adhesion of highly metastatic MDA-MB-231 breast cancer cells to brain endothelial monolayers and to enhance their transmigration through an *in vitro* BBB model [100]. It has also been shown that small cell lung cancer cell-derived placental growth factor activates the VEGFR1/Rho/ERK signaling pathway in cerebral endothelial cells, resulting in the disassembly of tight junctions and promoting transendothelial migration [101].

### 3.6. Proteases Involved in the Formation of CNS Metastases

Proteolytic enzymes may play a role in several steps of metastasis formation, including invasion at the primary tumor site, intravasation, extravasation and metastatic colonization. Different proteolytic enzymes have been implicated in brain metastasis formation and migration of tumor cells through blood-brain barrier endothelial cells. The proteases involved are matrix metalloproteinases (MMPs), serine proteases and heparanase. These enzymes are mainly secreted by tumor cells; however, endothelial cells or astrocytes might also be induced by tumor cells to express proteases.

#### 3.6.1. The Role of Matrix Metalloproteinases in the Formation of Brain Metastases

Several results suggest that MMP-2 plays a key role in the brain metastasis formation of breast cancer cells [102–104], melanoma [105] and leukemic cells [106]. MMP-3 and MMP-9 are also implicated in the development of breast cancer brain metastases [103]. In addition, MMP-1 and MMP-9 were shown to be overexpressed in brain-seeking breast cancer cells in comparison with bone-seeking variants [107], while expression of MMP-2 and MMP-9 was found to be upregulated in breast cancer cells treated with angiotensin II, probably contributing to the increased migration through brain endothelial cells [108]. MMP-9 was found to be overexpressed by brain metastatic lung adenocarcinoma cells [109].

Regarding transendothelial migration of tumor cells through the BBB, MMPs might have special importance, because TJ proteins can be targets of MMP degradation. MMP-induced disruption of TJs was shown to promote invasion of tumor cells into the CNS [106]. Interestingly, not only tumor cells themselves produce MMPs, but they are able to induce the expression of proteases in brain endothelial cells, e.g., MMP-2 was shown to be induced in brain endothelial cells after coculture with breast cancer cells [110]. In this study COX-2 and MMP-2 produced by cerebral endothelial cells was found to facilitate the extravasation of breast cancer cells across the BBB.

#### 3.6.2. Other Proteases

ADAM-9, a member of the “a disintegrin and metalloprotease” (ADAM) family was found to be overexpressed in highly brain-metastatic non-small cell lung cancer sublines in comparison with parent or highly bone-metastatic sublines [111]. In melanoma, plasmin was proved to be a key determinant of crossing of the BBB and formation of brain metastasis [112]. Moreover, we have shown that during migration of melanoma cells through the brain endothelium, tumor cells release large amounts of gelatinolytic serine proteases, including seprase. Inhibition of these proteases or silencing of seprase could significantly reduce the number of extravasating melanoma cells [54].

#### 3.6.3. Heparanase

Heparanase is an endoglycosidase, degrading heparan sulfate proteoglycans, being a critical mediator of tumor cell proliferation, angiogenesis, invasion and metastasis. This is achieved by remodeling of the extracellular matrix, releasing growth factors, chemokines, angiogenic factors, bioactive cell-surface heparin sulfate fragments and through non-enzymatic (signaling) activities [113]. Heparanase is considered a critical molecular determinant of brain metastasis [114] in melanoma [115] and breast cancer [116], but surprisingly high exogenous heparanase concentrations were shown to downregulate invasion of brain metastatic melanoma cells [117]. Besides tumor cells, astrocytes were found to produce heparanase as well, significantly contributing to the brain colonization of melanoma cells [118].

### 3.7. Signaling Pathways Involved in Tumor-endothelial Interactions in the Brain

In order to be able to respond adequately to extracellular stimuli, cerebral endothelial cells are equipped with a whole set of receptors and signaling molecules [119–121], reviewed in: [122]. This may contribute to a considerable heterogeneity of cerebral endothelial cells as well [123]. Transendothelial migration of tumor cells requires the active involvement of both tumor and endothelial cells. Understanding the pathways activated both in tumor cells and in endothelial cells will help to identify molecular targets for cancer therapy.

#### 3.7.1. Rho/Rac Signaling

During invasion of tissues and migration through vessel walls and ECM components, metastasizing tumor cells require increased motility, which is dependent on the remodeling of the cytoskeleton. In this respect, members of the Rho family small GTPases were shown to have an indispensable role by regulating the two major modes of tumor cell movement, characterized by mesenchymal and amoeboid phenotype. The mesenchymal type of tumor cell movement requires elevated Rac1 activation and reduced Rho/ROCK signaling and is characterized by elongated cell morphology, formation of large membrane protrusions and dependence on integrins and extracellular proteolysis. On the other hand, the amoeboid migration type mimics movement of leukocytes, with a rounded morphology and generation of Rho/ROCK-dependent actomyosin contractile forces [124–126]. Interplay between these two types of tumor cell motility was shown to regulate movement of tumor cells during invasion of the extracellular matrix; however, little is known about the behavior of tumor cells meeting other types of barriers, *i.e.*, during intravasation into circulation and extravasation into diverse tissues, including transmigration through the BBB. It has been shown that inhibition of ROCK decreases the migration of small cell lung cancer cells through the brain endothelium [127]. This effect, however, is not due to changes in tumor cell movement, since Rho/ROCK signaling, cytoskeletal reorganization and the concomitant changes of the tight junctions of endothelial cells were responsible for the decrease of transendothelial migration of tumor cells. This, in turn, highlights the importance of endothelial cells in the extravasation of tumor cells, clearly indicating that the endothelium forms not only a passive barrier for metastatic cells, but takes an active part in the process.

#### 3.7.2. Src Signaling

Src family members are known to participate in many aspects of tumor progression and metastasis. In tumor cells, Src kinase may participate in the promotion of mesenchymal and inhibition of amoeboid motility [128] and in the phosphorylation of N-cadherin and dissociation of β-catenin during transendothelial migration [88]. Src kinases are known to regulate interendothelial junctions and endothelial permeability as well [129,130]; therefore, it would be interesting to understand whether (brain) endothelial Src signaling is involved in metastasis formation.

#### 3.7.3. The PI3K-Akt-PTEN Pathway

The PI3K-Akt pathway is a crucial regulator of cell survival and proliferation, and increased PI3K activity has been reported in several cancer types. In a recent study, a novel inhibitor of downstream PI3K was found to effectively control metastatic growth of HER2 positive breast cancer cells in multiple organs and resulted in a significant proportion of mice free from brain and bone metastases [131]. In addition, brain metastases of melanoma were shown to have significantly higher levels of phosphorylated Akt and lower PTEN than lung or liver metastases [132]. This pathway was shown to be activated not only in brain metastatic melanoma cells, but also in brain endothelial cells coming in contact with melanoma cell-conditioned media, inducing increased endothelial cell proliferation and motility [133]. Moreover, the PI3K inhibitor LY294002 was shown to reduce the number of ECM-invading breast cancer cells in the presence of pulmonary microvascular endothelial cells [134]. It was also shown that melanoma cell-associated VE-cadherin breakdown in HUVECs was not sensitive to LY294002, whereas transendothelial migration of melanoma cells was reduced in the presence of the PI3K inhibitor [135]. However, inhibition of PI3K had no effect on the transmigration of small cell lung cancer cells through brain endothelial cells [127].

#### 3.7.4. Other Pathways Implicated in Brain Metastasis Formation, Transendothelial Migration or Invasion of Brain Metastatic Tumor Cells

In the search for the determinants of brain metastasis formation, a few other signaling pathways have been described, which might be involved in the migration of tumor cells through the BBB. Recently, overexpression of endothelin receptor B was shown to result in an increased incidence of spontaneous CNS metastases of melanoma [136]. In melanoma, TGF-β2 was found to be crucial as well, since its expression is indispensable for the formation of parenchymal metastases [137]. Stat3 activation was found to play an important role in angiogenesis, invasion and brain metastasis formation of melanoma cells through dysregulated expression of bFGF, VEGF and MMP-2 [105]. In breast cancer cells HER2 (EGFR2), positivity was found to contribute to brain metastatic colonization [138], the HER2 transfectants giving a significantly increased number of macrometastases. Taking into account that the BBB prevents the delivery of trastuzumab (a HER2 monoclonal antibody used for the treatment of mammary tumors), HER2 positive breast cancer patients have an increased risk of mortality caused by brain metastases. Besides HER2 positivity, breast cancers with increased risk to develop brain metastasis are more likely to be estrogen receptor negative, to express the basal cytokeratin CK5/6 and to overexpress EGFR [139]. Mutations in EGFR have been implicated in the pathogenesis of another brain metastasis-giving tumor type, the non-small cell lung carcinoma. An increased risk of progression in the CNS was found in advanced non-small cell lung cancer with EGFR mutations [140]. In lung adenocarcinoma, activation of the canonical WNT/TCF pathway through LEF1 and HOXB9 was identified as a key element of metastasis formation to brain and bone [141].

There are several signaling mechanisms implicated in the proliferation, invasion or migration of tumor cells, which—although not investigated so far—might be involved in the migration of tumor cells through the BBB. The multidrug transporter P-glycoprotein, for example, has been shown to mediate the invasion of melanoma cells. P-gp was shown to cooperate with CD44 through the activation of MAP-kinases, leading to increased MMP activities [142]. Moreover, the MAP-kinase signaling pathways (including the mutations in BRAF) are critical in the development and progression of primary and brain metastatic melanoma; however, their role in the transendothelial migration of tumor cells has not been documented. ERK1/2 was shown to be activated in brain endothelial cells coming in contact with melanoma cell-conditioned media [133]. In melanoma, another pathway, phospholipase C signaling, might also be important, since it was shown to be involved in melanoma cell-induced endothelial junction disassembly in HUVECs [135]. Other promising targets might be receptor tyrosine kinases, e.g., Axl, which is highly expressed in several tumor types, including brain metastatic tumors [143] and cerebral endothelial cells as well [144]. Axl confers aggressive tumor behavior, leading to dissemination and metastasis formation [145]; however, no data are available about the role of Axl in brain metastasis formation.

### 3.8. Role of Astrocytes in Brain Metastasis Formation

Astrocytes have an indispensable role in the maintenance of BBB properties of cerebral endothelial cells. Therefore, they support endothelial cells in impeding tumor cells from penetrating into the brain. On the other hand, astrocytes have a protective role for brain metastases. Reactive astrocytes immediately localize to individual breast cancer cells even before extravasation and continue to associate with metastatic cells during the transmigration process and throughout the growth of the lesions [51]. Reactive astrocytes induce the protection of tumor cells from chemotherapy through sequestration of calcium from the cytoplasm of tumor cells and by upregulating survival genes in tumor cells [146,147]. Moreover, astrocytes secrete soluble factors that stimulate the proliferation of tumor cells in the brain microenvironment. In this respect, neurotrophins have a special importance in supporting the growth of melanoma cells [148]. In addition, astrocytes were shown to induce proliferation of lung and breast cancer cells by producing IL-1β, TNF-α and/or IL-6 [149,150]. Since inflammation was found to foster proliferation, survival and migration of tumor cells [151] and metastatic cells are also able to use several similar transendothelial migratory mechanisms as leukocytes, it is tempting to speculate that these proinflammatory cytokines secreted by astrocytes might not only induce proliferation of tumor cells, but also support the transendothelial migration and formation of new metastatic colonies in the brain. Astrocytes also produce heparanase, which was shown to contribute to the brain-metastatic specificity of melanoma cells [118], and MMP-9, which can support invasion of tumor cells and release growth factors from the extracellular matrix [51].

## 4. Conclusions

Tumor cells meet a supportive environment in the brain parenchyma, protected from chemotherapeutics and antitumoral immune response and containing soluble factors favoring their survival and proliferation. It is not surprising, therefore, that brain metastases of malignant tumors have limited therapeutic options. Hence, it would be of crucial importance to prevent the formation of brain metastases. One of the possible strategies is to target the step of migration of metastatic cells through the blood-brain barrier. The mechanisms of this process are largely uncharacterized; however, besides preventing cancer cells from entering the brain, brain endothelial cells seem to also play a protective role for metastatic cells during extravasation. Understanding these mechanisms is indispensable to find targets of prevention of brain metastasis formation.
